# The Diagnosis of Cancer of the Uterus

**Published:** 1902-07-12

**Authors:** 


### HOSPITAL CLINICS AND MEDICAL PROGRESS.

### THE DIAGNOSIS OF CANCER OF THE UTERUS.

FULLY impressed as we must be, by the statistics of modern surgery, with the fact that for the successful treatment of cancer of the uterus everything depends upon early diagnosis, the question arises whether we possess any means by which this diagnosis can be arrived at with certainty at an earlier stage than would be possible were we to trust to the ordinary classical symptoms of the disease, and in this connection we would draw attention to the stress laid by Dr. Francis Haultain [1] upon microscopical examination of the debris removed from the suspected case. He says that although there are a few who still sneer at the microscope as a reliable means for the diagnosis of early malignant conditions, and pin their faith almost entirely to grosser clinical features, there can be little doubt but that by it, and it alone, in many cases can a decisive opinion be arrived at in the earliest stages, while even in more advanced cases it is of the utmost value as a corroborative.

In cervical disease two signs stand out pre-eminent from the clinical aspect: first, friability of the diseased tissue; secondly, its hæmorrhagic character. A curette, when applied to the suspicious area, will seldom fail to remove a considerable portion of friable tissue. This in itself is almost convincing, but it should be supplemented by submitting the material so removed to microscopical examination.

Free hæmorrhage on gentle touch is a sign of grave import, which demands investigation by means of the curette and the microscope, and it is to be noted that a history of hæmorrhage, of menorrhagia, or metrorrhagic character, associated with localised cervical disease demands the same kind of investigation, even though bleeding may not occur on digital examination, for an apparently healthy os externum may be present, with advanced malignant disease of the cervical canal.

Cancer of the body of the uterus is almost uniformly of the adeno-sarcomatous type, and clinically it differs from malignant disease of the cervix in being met with equally frequently in nulliparæ and parous women, whereas cancer of the cervix is almost entirely confined to those who have borne children. It may occur after or before the menopause, but much more frequently after. The symptoms in the initial stages are much more definite than those of cervical cancer, and consist mainly, Dr. Haultain says, in the early appearance of profuse menorrhagia with the subsequent development of metrorrhagia and free leucorrhœal discharge, while from the presence of the growth in the uterine body this organ on palpation feels distinctly enlarged. A cancer of the body of the uterus may be mistaken for subinvolution of that organ or for fibromyoma, and here again it is by the microscope alone that a differential diagnosis can be made. A portion of the growth is to be removed by the curette and submitted to a complete microscopical examination, or, better still, the cervix may be dilated and the uterine cavity explored by the finger. This will show the typical fungating outgrowth in the uterine cavity which can be scraped away and examined. With antiseptic precautions the operation of exploration of the uterine cavity after dilatation of the cervix can be safely undertaken, and certainly, without exception, says Dr. Haultain, should simple curetting fail to remove the symptoms of a case where hæmorrhage forms the prime factor, it is advisable to dilate and palpate without further delay or renewed attempts at curetting.

If the origin of chorio-epithelioma or deciduoma malignum from chorionic villi be accepted it forms a unique growth of a parasitic nature necessarily arising only after pregnancy. An interesting feature connected with its origin is the frequency with which it follows myxoma of the chorion. In the majority of cases the area of active growth is situated superficially in the uterus, and there forms a tumour which bulges into its cavity, and should this be the case the early symptoms—profuse hæmorrhage following upon the expulsion of the ovum—will closely simulate an imperfect abortion. The disease should be suspected if bleeding follow the expulsion of a vesicular mole or return after thorough curetting for imperfect expulsion of the ovum. Whenever this happens the uterine cavity should be thoroughly explored by the finger and the scrapings carefully examined microscopically. Dr. Haultain concludes by saying that as cancer of the uterus is a mortal disease in which early detection can alone give any prospect of cure, he would impress the importance of uterine hæmorrhage of metrorrhagic and post-climateric type. How often are the irregular hæmorrhages of the climacteric period discounted as being normal and treated by a placebo without a thought of local examination! "Should signs of hæmorrhage be present at this time a local examination should be made a routine practice . . . and if need be the uterus dilated and explored, or a portion of the suspicious area removed for microscopic investigation." Needless to say, unless the practitioner be thoroughly conversant with the modes of preparation and with the appearances presented by normal and morbid uterine structures, he should forward the scrapings to an expert.

Yet, notwithstanding all that Dr. Haultain says, we must remember that he is not entirely in accord with all authorities. So far as mere diagnosis is concerned, no one can doubt that Dr. Haultain is in the right. But other matters have to be taken into consideration. Difficult as it is to convey the infection of cancer from case to case, the infection of a patient's own tissues by her own cancer is the easiest thing in the world, and is, indeed, one of the principal causes of non-success in many operations for the removal of cancerous disease. Mr. Bland-Sutton [2] has lately laid special stress upon this point. He holds that some of the most thorough and complete vaginal hysterectomies have been followed by the most rapid and extensive recurrences. "This is due," he says, "to . . . cancer infection of the tissues injured in the course of the operation. Look at vaginal hysterectomy for cancer from any point of view, and the results can only be regarded as depressing. . . . In cancer of the body of the uterus I have had better results than in hysterectomy for cancer of the cervix; and the best consequences of all have followed abdominal hysterectomy for cancer of the body of the uterus. *The worst method of all is to*

*dilate the cervical canal to be quite sure that the disease is cancer, and then perform vaginal hysterectomy.* In such cases the cancer infection of the surrounding tissue gives rise to rapid recurrence, and such large masses of cancer grow on the pelvic peritoneum under these conditions as are rarely, if ever, seen when the disease is allowed to pursue its natural course." While early diagnosis is the key to the only hopeful treatment—namely, extirpation, and while the removal and microscopical examination of portions of tissue are evidently aids to early diagnosis, one must be careful not to injure the prospects of cure by too rigorous an attempt at diagnostic certainty. The patient of course always takes the individualistic view. She cares nought for averages, but asks for positive assurance as to the nature of her disease before she submits to operation. But if one has regard to the sum total of the good to be done by operative surgery, it may be better now and again to remove a uterus unnecessarily than to run any risks of diffusing the disease.

[1] Practitioner, June, 1902.
[2] Tumours Innocent and Malignant, 1901.

